# A Qualitative Study Exploring the Experience of Double-Duty Nurse Caregivers

**DOI:** 10.1177/01939459251348135

**Published:** 2025-06-18

**Authors:** Carolyn S. Phillips, Galilea Dupree, Jung Kwak, Megan C. Thomas Hebdon

**Affiliations:** 1 School of Nursing, The University of Texas at Austin, Austin, TX, USA

**Keywords:** well-being, caregivers, family caregiver, double-duty caregiver, caregiver burden, employment, nurses

## Abstract

**Background:**

Double-duty nurse caregivers manage caregiving responsibilities within their professional nursing roles and in personal family caregiving contexts. This dual role often leads to complex challenges and conflicting demands between caregiving roles. Nurses who are family caregivers experience both professional and personal caregiving burdens and strains.

**Objectives:**

This study explored the experiences of double-duty nurses, highlighting the unique challenges they encounter.

**Methods:**

This qualitative descriptive analysis explored the experiences of double-duty nurse caregivers through semi-structured interviews. Participants (*n* = 16) were recruited throughout the United States and interviewed via Zoom. Qualitative descriptive and thematic analyses were used to analyze the data.

**Findings:**

Interviews from 16 participants were analyzed. Participants described their roles as a blessing and a curse, highlighting the emotional toll and professional fulfillment associated with caregiving. Major themes included (1) the nurse of the family; (2) family and professional caregiving conflicts; (3) better nurses for patients and family caregivers; and (4) dual support needs.

**Conclusion:**

The study underscores the multifaceted challenges that double-duty nurse caregivers face and emphasizes the necessity for comprehensive support strategies. Interventions should address emotional resilience, financial stability, and access to supportive resources to mitigate burnout and sustain caregiving practices within the nursing profession. Nurses carrying both roles may need additional workplace, family, and community support to manage their caregiving roles.

Double-duty nurse caregivers are characterized as individuals who deliver care to people with chronic and life-threatening illnesses within their nursing profession and respective family caregiving settings. Often, they are placed at a conflicting intersection between providing their patients with high-quality care, addressing the needs of their family caregiving, and recognizing their needs as individuals.^1^ By way of their demanding commitments to their care recipient, double-duty caregivers render their boundaries between serving others and themselves obscure, thereby exacerbating stress within both family and healthcare contexts.^1,2^

## The Impact of Competing Demands on Double-Duty Nurse Caregivers’ Well-Being

In a survey conducted by the American Nurses Foundation, 70% of nurses reported prioritizing their patients’ needs as superior to their own, and 77% perceived their level of risk for stress within the workplace as significant.^3^ Although stress is inherent to the nursing role, the COVID-19 pandemic has worsened nurse strain, yielded moral distress, and increased exhaustion, ultimately contributing to a rise in nurses leaving the profession altogether.^4^

Most family caregivers are not able to relinquish their caregiving responsibilities as easily, given the personal nature of their role. More often, family caregivers sacrifice their needs to increase their ability to support their care recipient. However, as the subjective caregiving burden increases, so does the caregivers’ tolerance for overextending their caregiving abilities beyond rational means.^5^ As such, emotional health is adversely affected by the cost of prioritizing caregiving over personal affairs.^6^

Financially, family caregivers often bear the responsibility of managing their family member’s expenses, such as food, housing, insurance, and medical care, in addition to managing their own financial needs. Reduced job flexibility, foregone income, and out-of-pocket medical expenses all contribute to substantial financial risks for family caregivers.^7^ Financial strain further heightens the emotional vulnerability of caregivers. Family preferences to pursue palliative care over life-sustaining measures are sometimes motivated by the cost of medical services in the United States.^8^

Given that financial and emotional support intertwine to cultivate well-being for caregivers, collectively addressing these needs is imperative to diminishing the caregiving burden. The stress from the COVID-19 pandemic and its well-documented effect on exhaustion among nurses in general^4^ supports the need for further research within the double-duty caregiver population. Double-duty nurses uniquely experience a state of perpetual caregiving that impacts their ability to safely administer care and live healthily.^9^ Furthermore, as the population ages, the demand for family caregivers is increasing as well.^10^ Numerous studies have highlighted the challenges of family caregiving, yet less is known about the cumulative impact of double-duty caregiving on the person’s well-being, if these stresses increase vulnerabilities or additional risks for the caregiver, and what impact the double-duty roles have on their professional caregiving capabilities.

Pearlin’s Stress Process Model^11^ posits that individuals coping with primary stressors related to caregiving demands, such as the care recipient’s disability or behavioral concerns (eg, dementia-related behaviors), are part of a larger picture of factors influencing the well-being of the caregiver and the care recipient. This confluence of factors can include the caregiver and care recipient’s backgrounds and context, secondary role strains, and secondary intrapsychic strains contributing to health outcomes.

For double-duty nurse family caregivers, we posit that the primary stressors are related to both their family caregiving and nursing employment responsibilities. Having two caregiving roles may cause stress compression and increased conflict among secondary role strains and intrapsychic strains.^1,9^ For example, double-duty nurse caregivers have job-caregiving conflict, heavy emotional and physical caregiving responsibilities on the job and at home, limited time between work and caregiving, conflict with their own family, colleagues, patients, and patients’ families, financial challenges related to both family caregiving and work, and increased pressure as a family caregiver due to their nursing role.^9^ This may exacerbate health outcomes and require coping and social support that addresses both caregiving roles.^9^

## Purpose

The purpose of this qualitative descriptive analysis is to understand how double-duty nurse caregivers’ emotional and financial experiences are comparable and distinct to those of broad-family caregivers. The findings will be used to identify support needs for future intervention purposes.

## Methods

The study was a qualitative descriptive analysis of data from a cross-sectional multi-method study addressing the roles and needs of double-duty nurse caregivers. The study was deemed exempt by the University Institutional Review Board. Double-duty nurse caregivers could opt to complete the quantitative survey and qualitative interview after reviewing the study cover letter. The report of the quantitative results is under review. Survey completion utilized implied consent, and written consent was not obtained.

### Participants and Procedures

Double-duty nurse caregiver participants were recruited nationally through social media, professional contacts, and with assistance from Trialfacts,^12^ a study recruitment company that uses social media advertising and research participant databases for recruitment. Eligibility criteria for participants included: (1) 18 years of age or older, (2) English speaking, (3) employed as a nurse (Licensed Practical Nurse (LPN), Registered Nurse (RN), Advanced Practice Nurse (APN), nurse faculty, graduate nursing student) in the last 2 years, (4) currently providing 10 or more hours of care per week for an individual with a chronic illness, (5) have technology access for online surveys and Zoom interviews, and (6) have an e-mail address for receiving study information.

Recruitment materials included a study survey link with an eligibility survey, the study cover letter, and a quantitative survey. After completion of the quantitative survey (demographics, caregiving and nurse characteristics, burden, holistic well-being, and workplace productivity), participants could opt to be re-contacted to participate in a semi-structured interview by providing their e-mail address. Twenty-four participants agreed to participate in the semi-structured interviews. Of the 24 interviews, 8 were deemed fraudulent after data collection and excluded, leaving 16 interviews. A fraud protocol was implemented in collaboration with Trialfacts to ensure study rigor (Supplemental Appendix A). After 16 interviews, data saturation was achieved, thus no more participants were recruited. All participants who completed the interview were given a $40 gift card.

The interview guide (Supplemental Appendix B) was developed by the author team based on the literature and personal experiences as double-duty caregivers. Interviews were conducted via Zoom and focused on participants’ nursing roles, family caregiving roles, challenges and benefits of being a double-duty caregiver, and support needs. Methods to ensure privacy and confidentiality were the use of the Zoom audio recording only, transcription, and de-identification of interviews, and destruction of audio recordings once transcribed.

### Data Analysis

Semi-structured interviews were audio-recorded and transcribed. Interview data were analyzed using a qualitative descriptive and thematic approach. A qualitative descriptive approach was chosen because the focus of this study was exploratory in nature, with a goal to remain close to the data and participants’ narratives.^13^ Inductive and deductive thematic analysis^14^ using Dedoose 9.0.17 (SocioCultural Research Consultants, LLC, Manhattan Beach, CA, USA) were also applied to understand patterns across double-duty nurse caregivers’ stories. Interviews were inductively coded by the team. All interviews were coded by the team to identify major themes. Meetings were held throughout the coding process to promote alignment in coding and interpretation. Trustworthiness was maintained using an audit trail of team meetings, a data dictionary, coding decision-making, coder triangulation, researcher reflexivity, and debriefing during team meetings. Standards for Reporting Qualitative Research guidelines were followed.^15^

## Results

Participants (*n* = 16) primarily identified as female (*n* = 15), with 1 participant identifying as gender variant/non-conforming. The average age was 47.7 years (range: 27-61). Most nurses identified as white, but 5 participants identified as Black or African American. Most participants worked in the outpatient setting. Participants reported caring for children, parents, spouses, and friends (Table 1).

**Table 1.** Participant Characteristics (*N* = 16).

Characteristic	*n* (%)	Mean ± SD	Range
Participant age		47.67 ± 9.95	27-61
Nursing experience		20.94 ± 11.02	2-37
Gender identity			
Female	15 (93.8%)		
Gender nonconforming	1 (6.3%)		
Race			
Black or African American	5 (31.3%)		
White	11 (68.8%)		
Ethnicity			
Non-Hispanic or Latino	14 (87.5%)		
Hispanic or Latino	2 (12.5%)		
Marital status			
Married or partnered	12 (75%)		
Single or divorced	4 (25%)		
Nursing education			
Associate’s degree	3 (18.8%)		
Bachelor’s degree	2 (12.5%)		
Graduate degree	4 (25%)		
Other (licensed practical nurse, diploma)	7 (43.8%)		
Employment status			
Full-time	13 (81.3%)		
Other (part-time or per diem)	3 (18.8%)		
Practice setting			
Inpatient	4 (25%)		
Outpatient	9 (56.2%)		
Academia	2 (12.5%)		
Hospice	1 (6.3%)		
Income			
$0-$74 999	3 (18.8%)		
$75 000-$149 999	9 (56.2%)		
>$150 000	2 (12.5%)		
Prefer not to answer	2 (12.5%)		
Care recipient relationship^a^			
Spouse	9 (56.2%)		
Parent	1 (6.3%)		
Sibling	1 (6.3%)		
Child	3 (18.8%)		
Friend	2 (12.5%)		
Care recipient age		70.97 ± 17.03	18-85

a Some participants had multiple care recipients.

### It’s a Blessing and a Curse

Thematic analysis revealed a prominent meta-theme, “It’s a blessing and a curse.” This dual perspective was threaded throughout the stories nurses told about their experiences as double-duty caregivers. One participant specifically described double-duty caregiving as “a blessing and a curse” stating:

You know I have the knowledge and the background to know what questions to ask. . .how to be proactive instead of reactive. . .to be on top of, of somebody’s health. But, then again, you can’t turn it off. . . You go to work, and you come home, and then. . .on your days off you have to be a nurse again. (Participant 22).

The 4 sub-themes describing the meta-theme include: (1) the nurse of the family; (2) family and professional caregiving conflicts; (3) better nurse for patients and family caregivers; and (4) dual support needs.

#### The nurse of the family

Many participants described a greater pressure to care, to understand, and to be perfect because they are the nurses of the family. For example, 1 participant said, “Well, I think it’s like, ‘Well, you’re the nurse, so you know. I’m sure you’ve heard that before, right?’” (Participant 23). Participants described pressure to know and understand from both sides, their family members and the healthcare professionals caring for their family members.

Caregivers identified challenges related to their family caregiving situation, either general challenges or challenges specific to their double duty as a nurse/family caregiver. Like other family caregivers, participants described the strain on personal relationships, isolation, and feeling like they cannot contribute to life beyond work and caregiving. Participants also described the inability to switch off from caregiving responsibilities. One participant said, “it’s almost as if I’m double playing my role as a nurse because I’m a nurse both at the hospital and home. So, I am pretty much a nurse 24/7” (Participant 12).

Another challenge described was that technically, the nurse knew how to perform the “nursing task” needed, but the boundaries and expectations were different between the nursing role and the caregiving role. Furthermore, 1 described that they could push patients harder than they can push a family member. Specifically, they knew what the care recipient needed to do to improve, but if you push a family member that hard, it could negatively affect the personal relationship. For example, 1 person said, “I can be hard on my patients in the office, but I don’t have to live with them” (Participant 3).

Emotionally, participants described feeling valued and having a sense of purpose, but also feeling overwhelmed, spent, and burned out. One participant said, “I’m empty. I think I gave too much, so I’m just trying to work on me right now” (Participant 20). Another said, “Sometimes I just cry, like all day long. Yeah, that’s you know. You try to bring some brightness or some humor into it, but you just get so burnt out” (Participant 17).

The double-duty caregivers also described the rewards that come with being the nurse in the family. For example, because they were the nurse in the family, they automatically became the caregiver, which allowed them the opportunity to have a long goodbye to their family member. One participant said, “I think it’s positive that um I do get to care for her like they say it’s, it is a long goodbye” (Participant 1). Another participant described being able to provide care they feel the care recipient deserves. They said, “It’s like I said, I’d rather her get taken care of and know she’s getting taken care of. . . instead of being like reactive. ‘Well, why wasn’t this done?’” (Participant 22).

Participants also appreciated having insider knowledge that helped them navigate a complex system and better advocate for their family members. One participant said, “I probably feel more confident taking care of my mom, and when I talk to the doctors, you know. I know what they’re talking about. I know how to really advocate for her. Those are really the positives” (Participant 18). Another participant described the impact of having positive relationships with their care recipient’s healthcare providers.

That way, you know, I know a lot of the physicians and it was a little bit easier to navigate. I felt more comfortable as well just because it was familiar faces. Some. . .of the faces were familiar. I don’t work in the clinic so at least them knowing that I worked at [hospital name] was kind of like. . .you know “I’m your coworker.” (Participant 2)

#### Family and professional caregiving conflicts

The impact on employment was another concern for participants, with caregiving responsibilities affecting job performance, attendance, and career progression. For example, 1 participant said,

My family’s blowing up my phone asking a billion and 1 questions, and then doctors, and I’m like, I’m trying to focus. . .like I have a patient who is not doing well, like this is my job, this is my priority right now. I have to focus. (Participant 2)

Participants also expressed how they felt pressured by their employers to prioritize their work over their caregiving responsibilities, making them feel vulnerable when requesting leave or accommodations due to caregiving responsibilities and fearing repercussions at work. One participant said,

They just feel like you should be at work, no matter what that’s like, your first and foremost responsibility. And it is a responsibility, but I don’t, I don’t think it’s, it’s um, I don’t think it’s right to expect somebody to choose between taking care of their family member and going to work and worry about like, “Am I going to get in trouble at work?” (Participant 19).

Participants described that their workplaces can be supportive, but it often depended on their tenure (length of time working), relationships with colleagues, and family caregiving experiences of their colleagues. For example, 1 participant said, “Both of my bosses are incredibly supportive and. . .my main boss, he has gone through something similar with his mom so he can completely relate” (Participant 2).

Further, multiple participants remarked that they stayed in their jobs longer when management was supportive. One participant said,

I have an amazing boss. I really do. . .that’s the reason I’ve been, I’ve been there for about three years now. She’s luckily very um very flexible with me. . .that’s, that’s kind of a reason why I stayed where I stayed, because she’s very flexible. (Participant 22).

Another participant stated, “I think it’s been a little bit more accepted, because I’ve been such a loyal employee. . .and that’s probably why I don’t. . .move for another job. Another place might not know me as well. . .my heart, my work ethic” (Participant 14).

Participants described having job options with greater flexibility, which was a positive, but that they often came at the cost of reduced income, loss of career progression, and more frequent job changes. One participant said,

Yeah, I even had the opportunity to get a promotion, and I turned it down because I was like, “No, I can’t.” I would have gotten a pay raise, but it would have been five days a week, and I said, “No, because you know my with my three twelves, I can easily switch my day around.” If I need to, like, I need to come in another day. I can’t do that. Five days a week, Monday to Friday. There is no flexibility. (Participant 5)

Describing the financial impact of balancing their family caregiver role, 1 participant said,

I wouldn’t be able to pick up a shift that I wanted to, or um to miss out on some, you know, bonus opportunities, or something like that, more like missing work, and maybe I’d already used my PTO [paid time off]. So, I was not getting paid for those hours that I was gone, but I had to miss, and maybe I couldn’t make it up because there weren’t. . .any available opportunities to make it up for that week. (Participant 19)

Another participant said,

You know, you go from a nurse’s wage to a thousand dollars a month. It’s a culture shock. So, 1000 dollars a month, actually 950, to take care of my husband, the more you make the more you spend. And we have his disability check, and, but we struggle from month to month to, to make ends meet. So that’s why I’m actually talking to you today just to come up with a little extra funds. (Participant 20)

#### Better nurse for patients and their family caregivers

Participants expressed enhanced empathy for their patients and family caregivers based on the lessons they had personally learned as family caregivers. Further, they had first-hand knowledge about the different community resources that are available to pass on to patients and their families. For example, 1 participant said:

Also, my compassion I have for other people it’s like you know I discharge a patient, and their issues don’t stop right there. They’re going to continue, and you know it’s going to be a lot on the family’s shoulders so trying to being able to acknowledge that and provide that compassion and support to them of the things that okay, make sure you get this and that. There were these programs that I had never known existed, but now I know they exist so that’s been very positive. (Participant 2)

One participant described a greater perspective on care by describing the need for health care professionals to practice more holistically and to inquire about potential support deficiencies. She said,

You know, “what’s your income? Do you have enough food?” You know, they don’t ask those kinds of questions, you know. “Do you know who you have insurance with?” People don’t know if they have Medicare or Medicaid. (Participant 20)

Last, another participant described a deeper understanding of family caregiver needs based on the combined professional knowledge and personal experience. When discussing her mother’s care, she said,

She doesn’t make enough to live on her own and to get health insurance and that kind of stuff so that part is frustrating, and I think that’s where I would love to see change. . . whether you’re a caregiver, nurse, or in the field and a caregiver at home. I think it would be beneficial, period. Like, why can’t I have my parents on my insurance? (Participant 2)

#### Dual support needs

Caregivers described the supportive care resources they need to manage their caregiving roles. They expressed a need for structural support, including accessible community resources and respite care, and felt torn between prioritizing their own needs and those of their employer, patients, and care recipients. One participant said, “Not every day, but most days you feel like they’re the priority and they come first and then everything else is after that” (Participant 7).

Despite existing community support programs, accessing these resources proved challenging, particularly for those working nontraditional hours. One participant said, “I know there are community things out there but usually a lot of those are designed for people who are Monday through Friday, 9 to 5 kinds of people. . .Well, I’m on my way to work at six-thirty on Tuesday” (Participant 19).

Participants expressed interest in a specific space to talk to other double-duty caregivers. In general, most participants felt they had people to talk to, but that they would benefit from talking to other nurses who were going through the same thing and who understood the challenges they face. One participant said,

Well yeah you know, sometimes it’s not always easy to everyone who takes both roles, so if maybe there could be some sort of support group, where caregivers and nurses need to support each other. Sometimes you need someone, people who do the same things as you because you feel as if they hear you and they know what you are going through. So, it would be really great if there’s some sort of a support group or emotional support group from other caregivers and nurses. (Participant 4)

## Discussion

This study explored the nuanced experiences of double-duty nurse caregivers, individuals who balance caregiving responsibilities both in their professional roles as nurses and in their personal lives. The qualitative findings revealed a complex interplay of challenges, rewards, and support needs faced by these caregivers, shedding light on the distinctive burdens they encounter within the healthcare and family caregiving contexts.

Participants consistently articulated the dual nature of caregiving as both a blessing and a curse. This duality reflects the multifaceted experiences of nurses who navigate caregiving responsibilities in their professional and personal roles. The sentiment of “blessing” resonated through the fulfillment derived from providing care that is deeply informed by professional expertise. Participants expressed how their nursing background equipped them with the skills to advocate effectively and navigate complex healthcare systems for their loved ones. This insider knowledge enhanced their confidence in caregiving and facilitated a deeper connection with care recipients and their families. Conversely, the “curse” aspect underscored the challenges inherent in balancing the double-duty role. Participants described emotional exhaustion, burnout, and the inability to find a break from caregiving responsibilities. These findings are similar to studies with double-duty nurses caring for family members at the end of life.^16^

Participants in our study articulated emotional challenges associated with dual caregiving roles. They described difficulty disengaging from caregiving responsibilities, leading to emotional exhaustion and strain on personal relationships. These challenges resonate with findings that highlight the prevalence of anxiety, grief, and social isolation among family and double-duty caregivers.^17^ Similarly, the study by Noohi et al^18^ underscored the critical role of social support in alleviating caregiver stress, emphasizing the need for accessible resources tailored to the unique needs of double-duty caregivers. The demanding nature of double-duty caregiving, characterized by constant care and supervision, hinders caregivers from adequately addressing the consequences associated with their caregiving responsibilities.^19^

Despite these challenges, participants expressed profound rewards associated with caregiving roles. They described enhanced empathy, strengthened family bonds, and the opportunity for meaningful connections with care recipients. These positive outcomes align with other studies that described a sense of purpose and increased familial closeness as a few of the psychosocial benefits of family caregiving.^20^

Double-duty caregivers face challenges such as limited control over work availability, scheduling conflicts, and time constraints imposed by their caregiving commitments, which result in financial losses and decreased opportunities for career advancement.^21^ Financial strain was a concern among participants, reflecting the economic impact of caregiving responsibilities. This echoes previous studies highlighting the economic burden faced by family caregivers, who frequently bear out-of-pocket expenses for medical care and face reduced earning potential.^7,9^ A study done by the American Association of Retired Persons (AARP) found that over 70% of caregivers regularly contribute their own income to buffer the care costs of their recipients, averaging $7242 in expenditure yearly.^21^

Caregivers often face dilemmas in balancing work commitments with caregiving duties, fearing negative repercussions in the workplace.^22^ While some participants reported access to flexible job options, these often came at the cost of reduced income and limited career advancement opportunities. This echoes the findings by Lee and Zurlo, who explored the financial strain experienced by female caregivers.^23^ They found that female caregivers experience greater disparities in household income and career trajectories. This study underscores the need for supportive workplace policies that accommodate caregiving responsibilities without penalizing caregivers, aligning with recommendations from the AARP Public Policy Institute regarding financial hardships faced by family caregivers.^24^

Participants expressed the need for comprehensive support systems that address their unique caregiving challenges. From an employment perspective, participants emphasized the benefit of managerial support to help reduce the emotional and financial implications of family caregiving. Supportive managers who are willing to accommodate the needs of caregivers and empathize with the complex nature of their employees’ caregiving situation are essential to fostering a caregiving-friendly workplace environment.^25^ Moreover, these perspectives highlight the need for prioritizing both flexibility and adaptability when considering supportive measures in the workplace for double-duty caregivers. By championing these factors, it is possible to improve the perceived support experienced by double-duty caregivers and provide them with the necessary respite to mitigate the multidimensional implications of caregiver fatigue.

Participants also emphasized the importance of accessible community resources and respite care. However, barriers such as limited availability, affordability, and compatibility with nontraditional work schedules hinder access to these resources. This underscores the necessity for policy reforms and community-based initiatives that enhance the accessibility and inclusivity of support services for double-duty caregivers.^26^ Further, interventions that promote emotional resilience and coping strategies tailored to double-duty caregivers are critical. Participants emphasized the importance of spaces to discuss caregiving challenges with peers who share similar experiences, highlighting the need for tailored support programs. A recent systematic review also found that nurses find the greatest support when debriefing with each other.^27^

Finally, familial support may buffer the emotional impact of double-duty caregiving. Establishing boundaries between the family members and the double-duty caregiver can help address the imbalance and foster improved understanding between the primary caregiver and their family network.^28^ Active efforts from family members to provide practical assistance with household chores, meal preparation, respite care, and caregiving duties can empower the primary caregiver to engage in self-management activities that promote effective stress management.^29^ Through the establishment of a supportive environment within the family unit, double-duty caregivers can seek solace and actively participate in self-care activities, effectively diminishing the emotional strain associated with family caregiving.

### Limitations and Future Recommendations

This study is limited in its generalizability due to its small sample size and qualitative nature. Importantly, this was an exploratory study, and the meaningful findings suggest that future studies with quantitative methods and a larger sample size are warranted. Further, the sample in this study was relatively homogeneous, which decreases transferability. Future studies should aim for a more diverse sample. Longitudinal studies could further examine which groups of double-duty caregivers are more vulnerable to family and employment conflicts and how they affect their burden and burnout.

### Policy Implications

From a policy perspective, workplace policies must evolve to better support caregivers. Advocating for caregiver-friendly policies such as paid family leave, flexible working arrangements, and caregiver-specific benefits can safeguard nurses from negative repercussions related to caregiving responsibilities. Policies ensuring job security for nurses needing extended leave or reduced hours due to caregiving needs are essential.

## Conclusion

This study highlights the multifaceted experiences of nurses who navigate double-duty caregiving roles as professional and family caregivers. By highlighting the complex interplay of emotional, financial, and professional demands, the study underscores the imperative for tailored interventions and supportive policies. Addressing these multidimensional needs is essential to enhancing caregiver resilience, mitigating burnout, and promoting sustainable caregiving practices within the nursing profession.

## Supplemental Material

sj-pdf-1-wjn-10.1177_01939459251348135 – Supplemental material for A Qualitative Study Exploring the Experience of Double-Duty Nurse Caregivers Supplemental material, sj-pdf-1-wjn-10.1177_01939459251348135 for A Qualitative Study Exploring the Experience of Double-Duty Nurse Caregivers by Carolyn S. Phillips, Galilea Dupree, Jung Kwak and Megan C. Thomas Hebdon in Western Journal of Nursing Research
