# Supplementary material for: A Qualitative Study Exploring the Experience of Double-Duty Nurse Caregivers
Source: West J Nurs Res. 2025 Jun 18;47(9):836–43. doi: 10.1177/01939459251348135 (PMC12329145; doi:10.1177/01939459251348135)
Supplement: sj-pdf-1-wjn-10.1177_01939459251348135 – Supplemental material for A Qualitative Study Exploring the Experience of Double-Duty Nurse Caregivers [file sj-pdf-1-wjn-10.1177_01939459251348135.pdf]

Appendix A. Fraud Detection Plan During Data Collection

1. Add a Captcha to the survey.
2. For screening questions, require text entry for all questions.
3. Review survey entries for the following characteristics:
    a. Is over half of the survey incomplete? (1 point)
    b. Is the email address provided unusual (e.g. long string of numbers or nonsense letters)? (1 point)
    c. Did it take the participant less than 5 minutes to complete the survey? (1 point)
    d. Does the email match or closely match a prior submission? (1 point)
    e. Is there conflicting information in survey responses? (1 point)
    f. Are responses to questions incongruent or unclear? (1 point)

    Action: If score of 1, the participant receives an email listed under potentially fraudulent. If score of 2 or greater, the participant receives an email listed under fraudulent.

Additional Measures After Data Collection

1. Answers are inconsistent (no 0 points, yes 1 point)
2. Directly from TrialFacts after 9/27 (no, remove)
3. Interview fishy (no 0 points, yes 1 point)
4. Disclose they are not a nurse (remove)
5. Similar IP (4-5 digits) (no 0 points, yes 1 point) (exception: June 21-26-AANP Conference)
6. Identical IP Address (remove) (exception: June 21-26-AANP Conference)
7. Use nursing credentials that are given outside the US (remove)
8. Longitude/Latitude identical (remove) (exception: June 21-26-AANP Conference)
9. Longitude/Latitude similar (no 0 points, yes 1 points) (exception: June 21-26-AANP Conference)
10. Longitude/Latitude outside the United States (remove)
11. IP address quality check– suspicious IP (remove)
12. Contact info identical (remove)
13. Recaptcha score less than .9 (no 0 points, yes, 1 points)

    Action: If score of 2 or more, remove participant data.

Appendix B. Interview Guide

1. Tell us about your experience of being a nurse and a family caregiver.
2. Tell us about the challenging aspects of balancing your role as a nurse and a family caregiver.
3. Tell us about the positive aspects of being a nurse and a family caregiver.
4. Describe the impact your family caregiving role has had on employment.
5. Tell us about the financial implications of being a family caregiver.
6. Describe the emotional toll of balancing your role as a nurse and a family caregiver.
